# Brain Aging and APOE ε4 Interact to Reveal Potential Neuronal Compensation in Healthy Older Adults

**DOI:** 10.3389/fnagi.2018.00074

**Published:** 2018-03-20

**Authors:** Elisa Scheller, Lena V. Schumacher, Jessica Peter, Jacob Lahr, Julius Wehrle, Christoph P. Kaller, Christian Gaser, Stefan Klöppel

**Affiliations:** ^1^Department of Psychiatry and Psychotherapy, Faculty of Medicine, Medical Center-University of Freiburg, University of Freiburg, Freiburg, Germany; ^2^Freiburg Brain Imaging Center, University of Freiburg, Freiburg, Germany; ^3^Department of Neurology, Faculty of Medicine, Medical Center-University of Freiburg, University of Freiburg, Freiburg, Germany; ^4^Medical Psychology and Medical Sociology, Faculty of Medicine, University of Freiburg, Freiburg, Germany; ^5^University Hospital of Old Age Psychiatry and Psychotherapy Bern, Bern, Switzerland; ^6^Department of Medicine I, Medical Center-University of Freiburg, Freiburg, Germany; ^7^Berta-Ottenstein-Programme, Faculty of Medicine, University of Freiburg, Freiburg, Germany; ^8^German Cancer Consortium (DKTK), Freiburg, Germany; ^9^German Cancer Research Center (DKFZ), Heidelberg, Germany; ^10^BrainLinks-BrainTools Cluster of Excellence, University Medical Center Freiburg, Freiburg, Germany; ^11^Department of Psychiatry and Psychotherapy, Jena University Hospital, Jena, Germany; ^12^Department of Neurology, Jena University Hospital, Jena, Germany

**Keywords:** functional magnetic resonance imaging, BrainAGE, aging, APOE, neuronal compensation, working memory, multiple regression, moderator analysis

## Abstract

Compensation implies the recruitment of additional neuronal resources to prevent the detrimental effect of age-related neuronal decline on cognition. Recently suggested statistical models comprise behavioral performance, brain activation, and measures related to aging- or disease-specific pathological burden to characterize compensation. Higher chronological age as well as the APOE ε4 allele are risk factors for Alzheimer's disease. A more biological approach to characterize aging compared with chronological age is the brain age gap estimation (BrainAGE), taking into account structural brain characteristics. We utilized this estimate in an fMRI experiment together with APOE variant as measures related to pathological burden and aimed at identifying compensatory regions during working memory (WM) processing in a group of 34 healthy older adults. According to published compensation criteria, better performance along with increased brain activation would indicate successful compensation. We examined the moderating effects of BrainAGE on the relationship between task performance and brain activation in prefrontal cortex, as previous studies suggest predominantly frontal compensatory activation. Then we statistically compared them to the effects of chronological age (CA) tested in a previous study. Moreover, we examined the effects of adding APOE variant as a further moderator. Herewith, we strived to uncover neuronal compensation in healthy older adults at risk for neurodegenerative disease. Higher BrainAGE alone was not associated with an increased recruitment in prefrontal cortex. When adding APOE variant as a second moderator, we found an interaction of BrainAGE and APOE variant, such that ε4 carriers recruited right inferior frontal gyrus with higher BrainAGE to maintain WM performance, thus showing a pattern compatible with successful neuronal compensation. Exploratory analyses yielded similar patterns in left inferior and bilateral middle frontal gyrus. These results contrast those from a previous study, where we found no indication of compensation in prefrontal cortex in ε4 carriers with increasing CA. We conclude that BrainAGE together with APOE variant can help to reveal potential neuronal compensation in healthy older adults. Previous results on neuronal compensation in frontal areas corroborate our findings. Compensatory brain regions could be targeted in affected individuals by training or stimulation protocols to maintain cognitive functioning as long as possible.

## Introduction

Neuronal compensation as an individual's reaction to cognitive challenge with the aim to maintain cognitive performance has been increasingly investigated over the past years in healthy aging and beginning neurodegenerative disease. Several theoretical frameworks describe compensation as a flexible recruitment of additional neuronal resources when existing networks reach their capacity limits and are not sufficient anymore for successful cognitive performance (Cabeza, 2002; Davis et al., 2008; Park and Reuter-Lorenz, 2009; Stern, 2009; Reuter-Lorenz and Park, 2014). Together with these frameworks, a debate on the interpretation of increased brain activation as compensatory arose (Price and Friston, 1999; Friston and Price, 2003; Grady, 2012). Moreover, an increase in brain activation cross-sectionally might not be maintained longitudinally, but be transformed in an overall decrease in functional response over time (Nyberg et al., 2010). To overcome this debate, clear-cut criteria to unambiguously characterize increased brain activation in frontal cortex as compensatory have been published recently (Cabeza and Dennis, 2013). Cabeza and Dennis state that successful compensation is indicated by an increase in activation that is positively related with task performance. In addition, guidelines for a translation of such criteria to statistical models have been suggested (Gregory et al., 2017). To establish a statistical model of compensation, three components are necessary: task performance, a measure of brain activation as well as a measure related to pathological or disease burden for the condition investigated (Cabeza and Dennis, 2013; Gregory et al., 2017).

Measures of task performance and brain activation for compensation models can be derived from task-based fMRI experiments. Approximations of pathological burden specific to healthy aging or a certain neurodegenerative disease can be specified according to the group investigated. In patients with beginning neurodegenerative disease, regional brain volume of an area affected by the disease can be used, e.g., striatal volume in Huntington's disease (Klöppel et al., 2015; Gregory et al., 2017). Regarding healthy aging, chronological age (CA) is an easily available proxy of biological age or pathological burden due to aging and has been utilized in models of compensation before (Scheller et al., 2017), though CA is linear in nature and does not cover individual deviations from average aging. A more sophisticated measure covering such deviations is the brain age gap estimation (BrainAGE, Franke et al., 2010), as it takes into account individual heterogeneity of brain anatomy. BrainAGE is estimated from T1-weighted structural MRI scans with the help of kernel regression. After estimation, BrainAGE constitutes the deviation (in years) from an individual's CA. The measure has proved helpful as a biomarker predicting the conversion from Mild Cognitive Impairment (MCI) to Alzheimer's disease (AD) and even outperforms other established markers of disease progression (Gaser et al., 2013). Moreover, BrainAGE is significantly related to markers of poor health such as indices of the metabolic syndrome and kidney and liver function in healthy older adults (Franke et al., 2014), and thus seems to reflect pathological burden in great detail. Recently, BrainAGE has proved a viable biomarker for aging, as individuals with older-appearing brains showed an increased mortality risk (Cole et al., 2017). On the other hand, younger-appearing brains seem to be related to higher years of education and the number of flights of stairs climbed daily, i.e., physical exercise (Steffener et al., 2016).

The current study is the first to combine BrainAGE with functional imaging data and to implement BrainAGE as a proxy for pathological burden in a model of neuronal compensation. Hence, we aimed at linking changes in brain activation to an underlying structural correlate (Gregory et al., 2017). This study builds on recent work on the same sample, where CA was successfully combined with APOE variant as moderator variables in a multiple regression model to unequivocally detect successful compensation in older adults at risk for neurodegenerative disease (Scheller et al., 2017). Carrying the APOE ε4 allele is an established risk factor for sporadic AD (Corder et al., 1993; Farrer et al., 1997). In a previous study, we showed that ε4 carriers activated medial frontal and inferior frontal areas to a greater extent compared to non- ε4 carriers during a working memory (WM) task, pointing to successful compensation in genetically burdened individuals. These effects were not additionally moderated by CA. Therefore, in the current study we strived to investigate if BrainAGE might aid to reveal additional compensatory areas in the same sample. In longitudinal data, BrainAGE changed to a greater extent in APOE ε4 carriers compared to non-carriers (Löwe et al., 2016). Thus, it is of great interest to examine a combination of both biomarkers within one model of compensation.

In the present experiment, we restricted our search for compensatory brain activity to prefrontal cortex, as previous studies of WM and APOE variant suggest potential compensation predominantly in prefrontal areas (Filbey et al., 2006, 2010; Wishart et al., 2006; Chen et al., 2013). Reviews of WM function in healthy older adults as well as early neurodegenerative disease corroborate these findings (Cabeza and Dennis, 2013; Reuter-Lorenz and Park, 2014; Scheller et al., 2014). Several behavioral and structural imaging studies point to an association between WM function and APOE allele status, as WM and other frontal cognition deficits in older APOE ε4 carriers were detected (Reinvang et al., 2010; Caselli et al., 2011; Bender and Raz, 2012; Greenwood et al., 2014). Moreover, frontal areas such as the dorsolateral prefrontal cortex (DLPFC) are investigated as targets for noninvasive brain stimulation (NIBS), e.g., transcranial direct current stimulation (tDCS) combined with cognitive training (Flöel, 2014; Jones et al., 2017; Ruf et al., 2017). In the future, tailoring stimulation to compensatory areas could constitute an approach to maintain cognitive abilities as long as possible (Scheller et al., 2014).

Taken together, the aim of the current study was to investigate compensatory recruitment in healthy older individuals to maintain WM function. First, we hypothesized that individuals with higher BrainAGE might require additional neuronal resources to perform a cognitively demanding WM task successfully. To this end, the moderating effect of BrainAGE on the relationship between task performance and brain activation was investigated with multiple regression with interaction effects. This assessment was compared to previously published findings of the same sample with CA instead of BrainAGE as a moderator (Scheller et al., 2017). Second, we examined the additional burden by the APOE ε4 allele on the relationship between task performance and brain activation and therefore implemented APOE variant as a second moderator variable. We hypothesized that the combined burden of a higher BrainAGE and the ε4 allele might reveal an augmented need for compensation in prefrontal cortex. With these analyses, our approach aimed at yielding further insight into differential compensatory mechanisms depending on the respective measure of pathological burden in healthy older individuals.

## Materials and methods

### Sample

Thirty-four community-dwelling healthy older adults (20 females, mean age 68.82 years, *SD* 5.33, range 61–80) were recruited as part of a project investigating neuronal plasticity in aging and early neurodegeneration at the University Medical Center Freiburg. All were right-handed, had normal or corrected-to-normal visual acuity and no history of psychiatric or neurological disease as well as adequate performance in a sensitive cognitive test (Montreal Cognitive Assessment [MOCA] score ≥24; Nasreddine et al., 2005). We chose the MOCA cut-off at 24 to reduce the number of false positive exclusions (Luis et al., 2009; Roalf et al., 2013). The local Ethics Committee approved the study and all participants gave written informed consent prior to participation.

### BrainAGE estimation and APOE genotyping

BrainAGE of each participant was estimated based on the individual T1-weighted anatomical image with a voxel size of 1 mm^3^ and respective CA. A detailed description of the algorithm can be found in previous publications (Franke et al., 2010; Gaser et al., 2013). We implemented the BrainAGE measure based on both gray and white matter to incorporate the entire brain structure in our model. In short, relevance vector regression (RVR) is utilized as statistical framework. The model is trained with the help of structural imaging data of a training sample, in this case we used 547 subjects of the IXI database (http://brain-development.org/ixi-dataset/). Then, the BrainAGE index of all participants was estimated using individuals' segmented T1-weighted images that were derived using the SPM8 package (http://www.fil.ion.ucl.ac.uk/spm) and the VBM8 toolbox (http://www.neuro.uni-jena.de/). We used the affine registered segmentations for gray and white matter that were smoothed with a 4-mm full-width-at-half-maximum smoothing kernel. After smoothing, data were resampled to 8 mm and a data reduction was performed by applying principal component analysis (PCA), utilizing the “MATLAB Toolbox for Dimensionality Reduction” (https://lvdmaaten.github.io/drtoolbox/). PCA was only performed on the training sample and the estimated transformation parameters were subsequently applied to the test sample. The BrainAGE score indicates the deviation (in years) from an individual's CA. A positive BrainAGE score implies an older-appearing brain (Cole et al., 2017), whereas a negative score signifies that the respective individual's brain is younger-appearing. The score can be added to CA to directly compare both measures (**Figure 2**), e.g., the BrainAGE of +2.5 of an individual with a CA of 79 yields a value of 81.5 years. In the current manuscript, we use “BrainAGE” to label the deviation from CA, and “estimated BrainAGE” to label BrainAGE + CA in years.

Genotyping of the sample is described in detail in previous work (Scheller et al., 2017). Twelve participants were identified as ε3/ε4 heterozygotes, 17 as ε3/ε3 homozygotes, two as ε2/ε3 heterozygotes and three as ε2/ε2 homozygotes. The allele frequencies in our sample reflect the distribution in the German population (~8% ε2, ~78% ε3, and ~14% ε4; Corbo and Scacchi, 1999). As subsample sizes did not allow for further stratification, we decided to classify APOE genotype as a dichotomous variable (12 ε4 carriers, who were all ε3/ε4 heterozygotes, and 22 non-ε4 carriers) for our statistical models. A classification of all participants according to their allele status as well as their BrainAGE index can be found in Supplement 1.

### Verbal n-back task and behavioral data

The blocked verbal n-back task consisting of 0-, 1-, and 2-back conditions has been described in detail in our previous manuscript (Scheller et al., 2017). In short, participants lay supine in the scanner while viewing instructions and stimuli via a mirrored projection system. They responded with the index and middle finger of their right hand using a custom-built 2-button response box. After a 5 s instruction screen, letters were presented one at a time for 1,500 ms each with 1,000 ms blank screen inter-stimulus interval (ISI), while subjects had to indicate by pressing a button with their dominant index finger whether the currently presented letter was the same as the previous (1-back) or second-last (2-back) letter. If the current letter was not the same, they had to indicate this by pressing a button with their middle finger. A third condition (0-back) served as a baseline to contrast against 1-back and 2-back and did not include working memory load. Here, either the letter A or B was presented for 1,500 ms each with 1,000 ms ISI and subjects had to press with their index finger if the current letter was A and with their middle finger if it was B. The three conditions (2-back, 1-back, 0-back) were presented in blocks of 10 letters in a pseudo-randomized order. There were six blocks per condition resulting in 60 trials per condition and each block lasted 30 s including 5 s of instructions. As an index of task performance, we computed accuracy as percentage of correct responses for each condition as well as reaction times as the latency between stimulus display and corresponding button press. Compatibility with the normal distribution was tested using Kolmogorov–Smirnov tests for all performance indices. To assess changes in performance between low and high WM load across the whole sample, we computed paired *t*-tests or Wilcoxon signed-rank tests where appropriate for accuracy and reaction times. In addition, performance indices were compared between ε4 and non-ε4 carriers with independent *t*-tests to ensure that identified brain activation differences were not caused by a performance deficit. For the imaging data analyses, accuracy as percentage of correct responses was used as measure of task performance.

### Imaging data

#### Data acquisition and processing

Detailed descriptions of imaging data acquisition as well as preprocessing procedures can be found in a previous study of the same sample (Scheller et al., 2017). Preprocessing and subsequent statistical analyses were performed with the Statistical Parametric Mapping software (SPM8 r4667; Wellcome Trust Centre for Neuroimaging, http://www.fil.ion.ucl.ac.uk/spm). In short, functional images were coregistered to respective anatomical scans, which were segmented with the VBM 8 toolbox (r435; http://dbm.neuro.uni-jena.de/software/). First-level analysis was conducted using a general linear model (GLM) approach in native space. Normalization took place before 2nd level analyses, such that images were resampled to a spatial resolution of 1.5 cubic mm and smoothed with a 6 mm full width at half maximum (FWHM) Gaussian kernel. For group multiple regression analyses, contrast images of interest comparing 2-back vs. 0-back conditions were entered in a multiple regression model. Activation peaks were anatomically labeled with the Anatomy Toolbox for SPM (version 1.8, http://www.fz-juelich.de/inm/inm-1/DE/Forschung/_docs/SPMAnatomyToolbox/SPMAnatomyToolbox_node.html, see e.g., Eickhoff et al., 2005). All group analyses were computed voxel-wise but restricted to a region of interest that comprised the entire prefrontal cortex due to the predominantly frontal location of compensatory areas in previous studies (see section Introduction). To this end, we defined an anatomical mask with the WFU pickatlas toolbox (Maldjian et al., 2003) comprising all bilateral frontal cortical areas (including the insular cortex).

#### Multiple regression analysis

To investigate whether BrainAGE and APOE variant significantly moderate the relationship between task performance and brain activation and thus to draw conclusions on neuronal compensation, we chose multiple regression as the favorable statistical model. Multiple regression is a simplification of the model suggested for longitudinal data by Gregory et al. (2017). Compared to the often-found group analyses in the field of neuroimaging, multiple regression is advantageous, as the sample can be investigated in a continuous fashion instead of being artificially dichotomized. Moreover, the possibility to introduce moderator variables in multiple regression enables investigation of interaction effects across the whole continuum represented by the sample (for introductions, see Cohen et al., 2003; Jaccard and Turrisi, 2003; Hayes, 2013). Hence, we allowed performance, BrainAGE and APOE variant to interact in the prediction of brain activation to investigate potential compensation.

**Figure 1:**
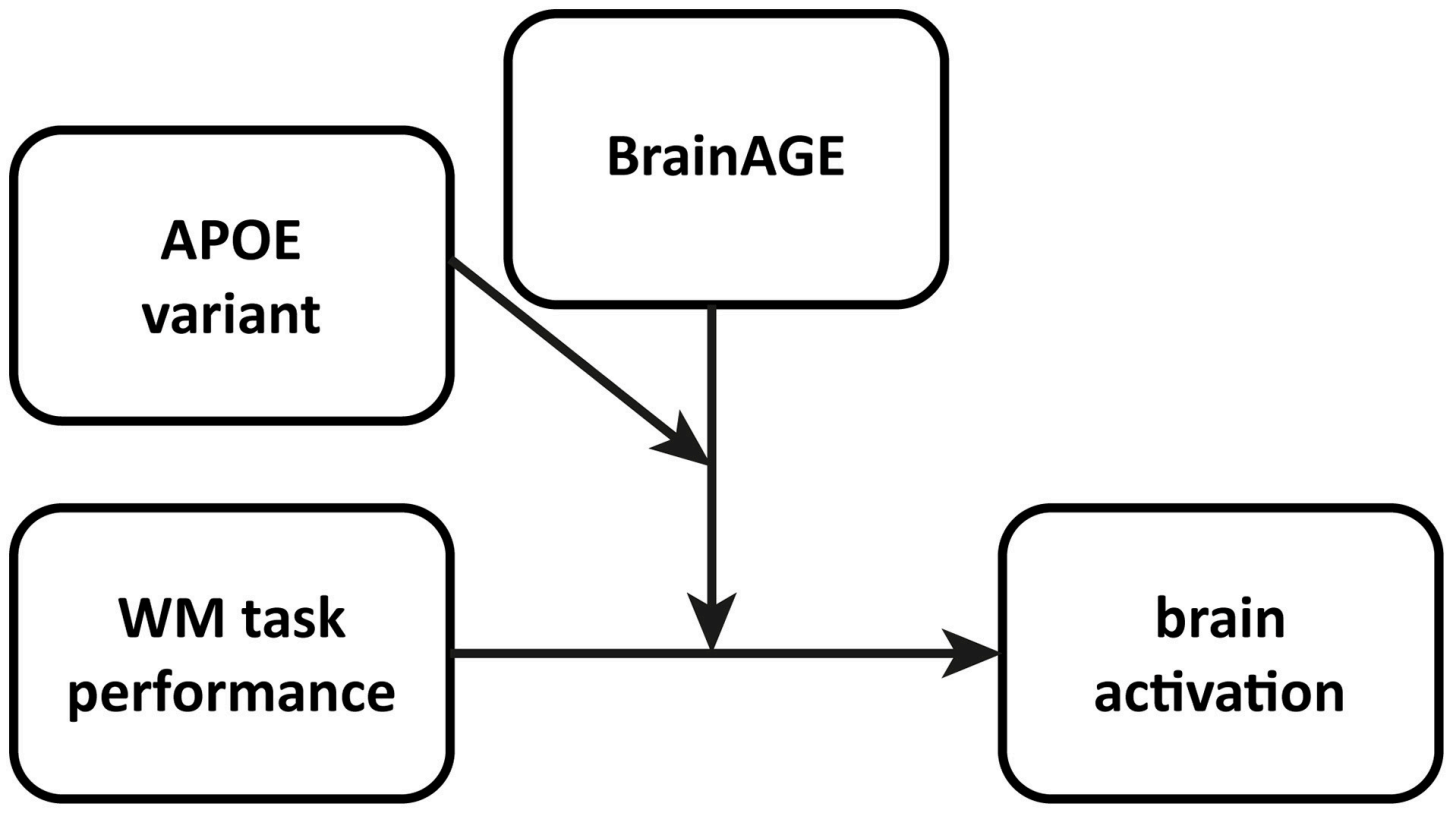
Conceptual diagram of the multiple regression model employed to analyze interaction effects in fMRI data allowing for moderated moderation. WM performance is the “focal” predictor, whereas BrainAGE as well as APOE variant act as moderators. In this model, 2- as well as 3-way interactions are possible, so as the focal predictor and both moderators can interact with each other. See also model templates for PROCESS Macro, http://www.afhayes.com/.

To assess the moderator effects of BrainAGE and APOE variant, we defined a multiple regression model for the 2-back condition of our n-back task (Figure 1). As expected, the 1-back condition showed a ceiling effect (Table 1), thus not offering sufficient variability for meaningful further interpretation. To construct the multiple regression model, we entered 2-back accuracy as “focal” predictor, i.e., the primary predictor of interest and both BrainAGE and APOE variant (ε4 vs. non-ε4) as one continuous and one dichotomous moderator, respectively. The contrast image 2-back > 0-back constituted the dependent variable or outcome in both models. The resulting regression models contained behavioral performance, BrainAGE and APOE variant as well as all possible product terms for two-way and three-way interactions of these variables as regressors. To control for confounds, gender and years of education were entered as nuisance variables. Results from such multiple regression models with interaction effects are conditional on the specific centering of the predictors. Therefore, all continuous predictors were mean-centered prior to entering the model to yield meaningful results representing average sample characteristics (cf. Jaccard and Turrisi, 2003).

**Table 1:** Demographics, neuropsychological, and behavioral n-back data.

	**CA**			**BrainAGE**			**Years of education**			**MOCA**			**Accuracy (%)**									**Latency (ms)**								
					**0-back**			**1-back**			**2-back**			**0-back**			**1-back**			**2-back**		
	**ε4 (*n* = 12)**	**Non-ε4 (*n* = 22)**	**All (*n* = 34)**	**ε4**	**Non-ε4**	**All**	**ε4**	**Non-ε4**	**All**	**ε4**	**Non-ε4**	**All**	**ε4**	**Non-ε4**	**All**	**ε4**	**Non-ε4**	**All**	**ε4**	**Non-ε4**	**All**	**ε4**	**Non-ε4**	**All**	**ε4**	**Non-ε4**	**All**	**ε4**	**Non-ε4**	**All**
Mean	67.75	69.41	68.82	-0.07	-0.67	-0.46	14.58	14.86	14.82	26.50	27.23	27.00	98.14	99.30	98.98	86.42	93.32	98.80	75.33	79.91	78.29	613	620	618	797	819	811	968	983	978
*SD*	6.72	4.47	5.33	4.62	3.92	4.12	2.88	3.47	3.26	1.38	2.20	1.98	2.00	1.12	1.50	14.54	7.68	1.92	16.63	13.33	14.50	126	81	98	211	149	171	278	182	217
min	61.00	61.00	61.00	-4.70	-8.29	-8.29	10.00	7.00	7.00	24.00	24.00	24.00	95.00	97.00	95.00	60.00	68.00	60.00	42.00	38.00	38.00	467	478	467	493	622	493	623	614	614
max	80.00	75.00	80.00	10.00	6.84	10.00	18.00	20.00	20.00	28.00	30.00	30.00	100.00	100.00	100.00	100.00	100.00	100.00	92.00	98.00	98.00	893	850	893	1,184	1,186	1,186	1,508	1,342	1,508
p^*^	0.39			0.69			0.81			0.31			0.12			0.23			0.39			0.84			0.73			0.85		

*The last row contains p-values resulting from independent t-tests or Mann–Whitney U-tests assessing differences between ε4 and non-ε4 carriers*.

CA, chronological age; MOCA, Montreal Cognitive Assessment;

* *p represents the p-values after the assessment of differences between ε4 and non-ε4 carriers*.

All multiple regression analyses were conducted voxel-wise with the in-house developed IFX Toolbox for SPM8 (Kaller et al., 2012) based on the assessment of interaction effects as described by Jaccard and Turrisi (2003) and Bauer and Curran (2005). The toolbox identifies activation peaks showing significant interaction effects. We assessed respective interaction contrasts within prefrontal cortex at a voxel level threshold of *p* < 0.05 family wise error (FWE) corrected for multiple comparisons, paralleling previous work (Scheller et al., 2017). For further exploratory analyses, we applied an uncorrected threshold (*p* < 0.001).

Subsequently, the signal of significant peaks was extracted using the volume of interest function in SPM and further analyzed with the PROCESS macro version 2.16 in SPSS (Hayes, 2013) with a model allowing for moderated moderation (PROCESS Model 3). PROCESS and the IFX toolbox are based on the same literature and use equivalent implementations of multiple regression with interaction effects. PROCESS allows for further examination of interaction effects, e.g., the addition of more covariates. Hence, we double-checked and further characterized interaction effects with PROCESS. To be able to directly compare BrainAGE and CA in one regression model, we added CA as a nuisance variable to the model with task performance, BrainAGE, and APOE variant as predictors. We decided against operationalizing CA as a fourth moderator due to sample size constrictions and because CA was not identified as a significant moderator in previous work on the same sample (Scheller et al., 2017).

To visualize interactions, we used the Johnson–Neyman technique (Johnson and Neyman, 1936; Bauer and Curran, 2005; Hayes, 2013) considering the conditional effect of task performance on brain activity across the whole range of the moderator variables (see e.g., Kaller et al., 2015; Scheller et al., 2017 for examples with neuroimaging data). With this approach, it is possible to compute regions of significance within confidence bands for the moderator variable, i.e., BrainAGE ranges for which task performance significantly relates to the activation of certain brain regions.

## Results

### Behavioral data and BrainAGE estimation

**Figure 2:**
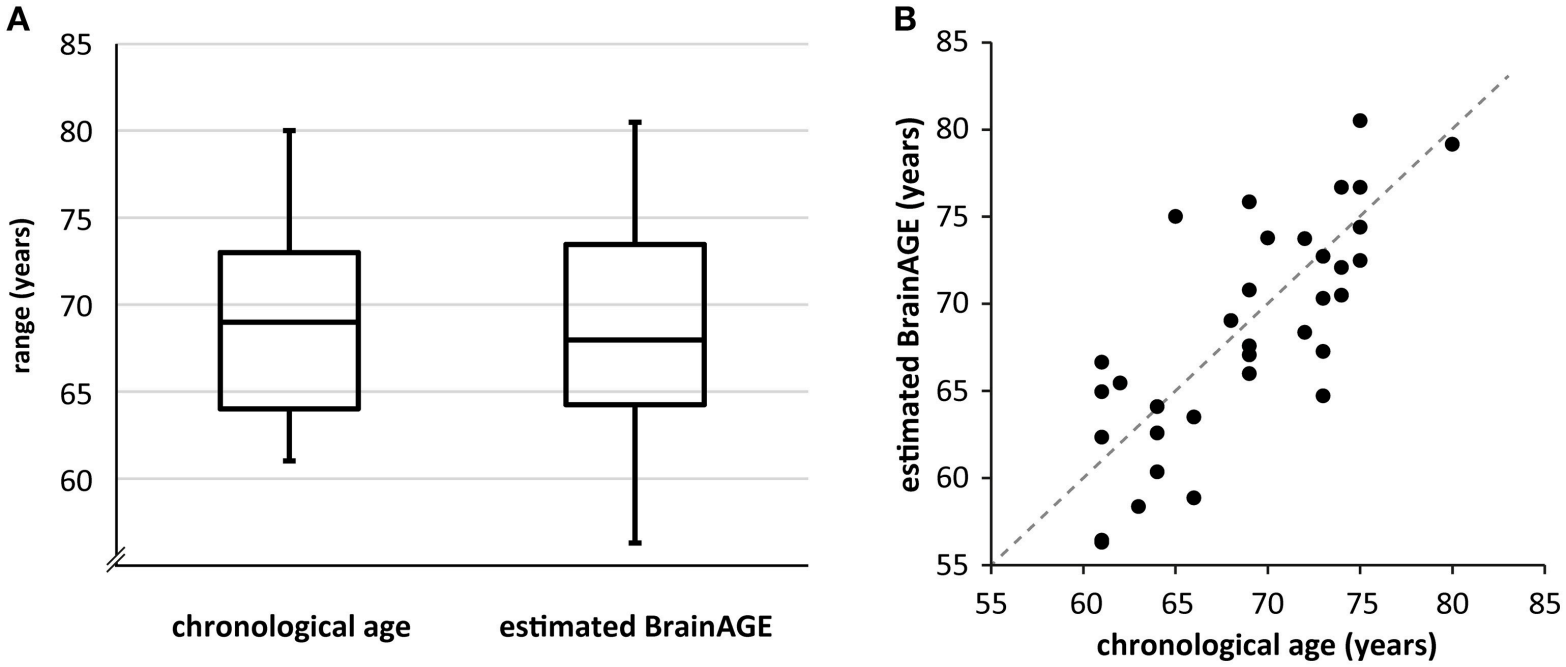
**(A)** Boxplot of CA and estimated BrainAGE across the sample. The BrainAGE score was added to CA for immediate comparability. The line within the boxes represents the median, the outer lines of the boxes depict the first and the third quartile, respectively. The error bars reach from the first and third quartile to the respective extrema. Note that estimated BrainAGE shows higher variability than CA as well as a trend toward lower values. **(B)** Scatterplot of CA and estimated BrainAGE. The angle bisector represents the line where CA equals estimated BrainAGE. Please note that the data point that is situated directly on the angle bisector belongs to a participant with a positive BrainAGE of 0.09.

Participants performed well across all task conditions. Ceiling effects were present in the 0-back and 1-back conditions. The 2-back condition showed high variability in performance (Table 1). BrainAGE and 2-back performance as well as their residuals were compatible with a normal distribution as confirmed by Kolmogorov-Smirnov tests (statistical threshold *p* < 0.05), hence fulfilled prerequisites of multiple regression. CA, BrainAGE as well as task performance in all conditions did not differ between ε4-carriers and non-carriers (Table 1). BrainAGE and CA were not significantly correlated (Pearson's *r* = −0.09, *p* = 0.60) as well as BrainAGE and task performance (*r* = 0.01, *p* = 0.95). Of the 34 participants, 14 obtained a positive BrainAGE index pointing to accelerated atrophy; 20 participants obtained a negative BrainAGE index and thus were estimated as younger than their CA (Figure 2, Supplement 1). Thus, there was a trend toward younger-appearing brains in the sample (Supplement 1). This reflects the neuropsychological characterization as cognitively intact older adults and potentially the high level of education (Table 1). Further analyses of task performance data can be found in a recent study of the same sample (Scheller et al., 2017).

### Imaging data: multiple regression

#### Task performance as focal predictor, BrainAGE vs. CA as a moderator

The main effect of task for the 2-back condition represented the well-known frontoparietal WM network (Owen et al., 2005). For an overview of MNI coordinates and images, please see Scheller et al. (2017).

We did not identify significant interaction effects of BrainAGE and task performance on activation in PFC. Hence, BrainAGE cannot be considered a moderator of the relationship between performance and brain activation such that individuals with higher BrainAGE need to recruit additional frontal regions to maintain performance. Similarly, CA did not moderate the relationship between performance and brain activation significantly, as shown in previous work. Thus, compensatory activation could not be detected.

#### Task performance as focal predictor, BrainAGE vs. CA and APOE variant as moderators

**Figure 3:**
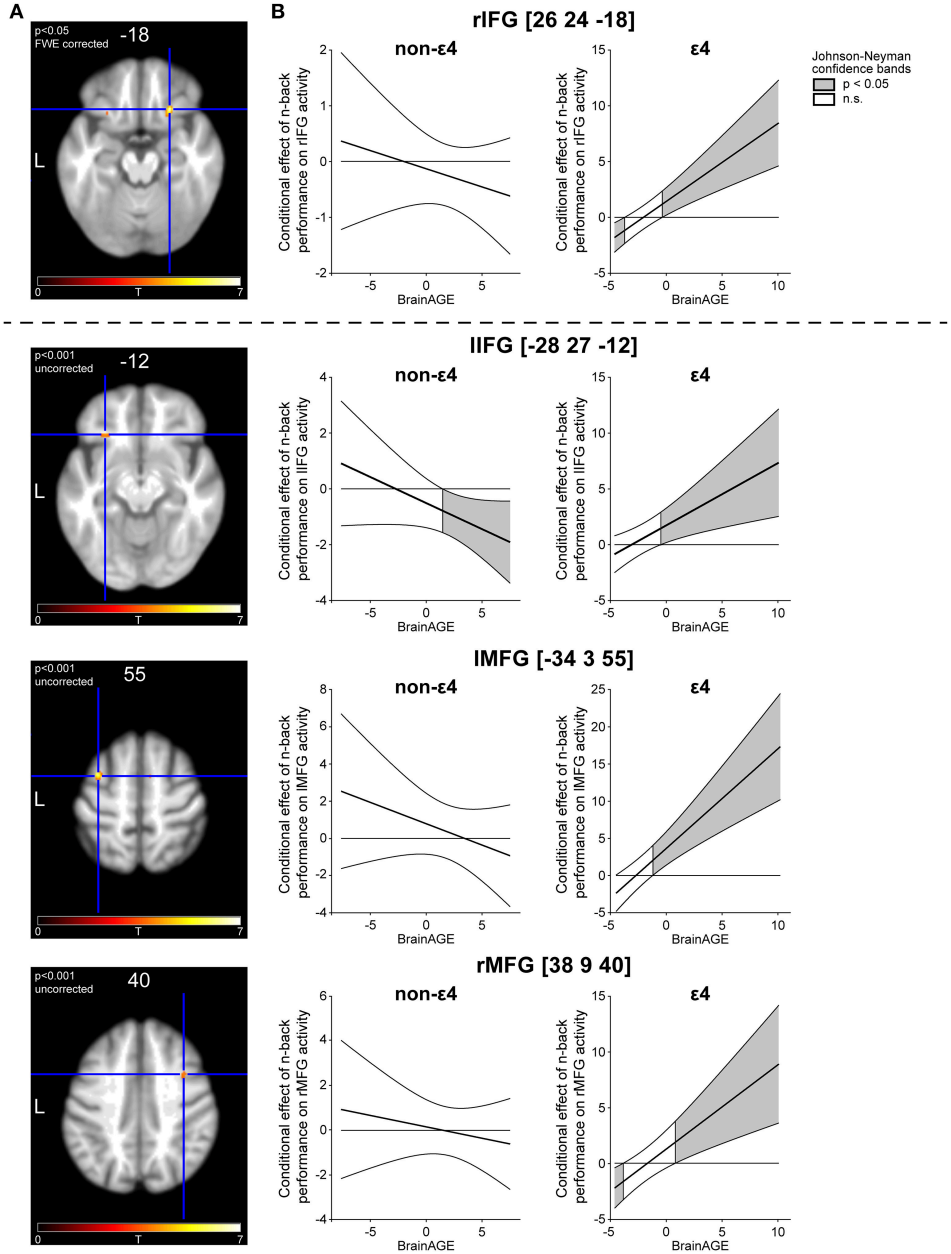
Three-way interaction of task performance, BrainAGE and APOE variant. **(A)** Activation peaks in bilateral inferior frontal as well as in bilateral middle frontal gyrus. Z-coordinates of the respective peak are depicted above the slices. **(B)** Johnson–Neyman (JN) confidence bands depicting the relationship of task performance and brain activation as a conditional effect across the whole range of the first moderator variable BrainAGE. To visualize the three-way interaction, JN bands are depicted separately for the second moderator APOE variant (ε4 vs. non- ε4).

After inspecting the above-described two-way interactions of performance and BrainAGE, we tested the three-way interaction of performance, BrainAGE, and APOE variant (ε4 vs. non-ε4) to determine if the addition of genetic burden as a moderator would reveal compensatory effects. Indeed, we found a significant three-way interaction in right inferior frontal gyrus pars orbitalis at *p* < 0.05 FWE corrected (rIFG; MNI x = 26 y = 24 z = −18; T = 6.91; cluster extent k = 50; *R*^2^ = 0.82; *p* < 0.001). The *R*^2^ increase due to the inclusion of the three-way interaction of task performance, BrainAGE, and APOE variant (*R*^2^-change) was 0.29, *p* < 0.001. The effect size Cohen's *f*^2^ of 1.61 was large according to the guideline defining *f*^2^ ≥ 0.02, *f*^2^ ≥ 0.15, and *f*^2^ ≥ 0.35 as small, medium and large effects, respectively (Cohen, 1988). Individuals carrying the ε4 allele recruited this region to a greater extent with increasing BrainAGE as can be derived from the dark-gray regions of significance within the Johnson–Neyman confidence bands (Figure 3B). The positive region of significance starts at positive BrainAGE values, which can be seen on the x-axis (Figure 3B, right column), hence the effect is present in individuals with increased atrophy (Figure 3B right column). Moreover, the slope of the line for ε4 carriers is positive and the region of significance is situated above the x-axis, signifying an increase of brain activation with better performance. Altogether, they showed activation compatible with successful neuronal compensation according to the criteria by Cabeza and Dennis (2013), while this effect was not present in non- ε4 carriers, as can be derived from missing regions of significance (Figure 3B, left column).

We further investigated the activation peak in rIFG with the PROCESS macro for SPSS. To be able to discern the incremental variability of using BrainAGE instead of CA as a moderator, we implemented CA as an additional nuisance variable. BrainAGE as a predictor significantly contributed to the model [unstandardized regression coefficient *b* = −0.01, standard error (SE) = 0.02, *t* = −2.12, *p* = 0.05], which was not the case for CA (*b* = 0.002, SE = 0.005, *t* = 0.48, *p* = 0.64). The three way interaction of task performance, BrainAGE, and APOE variant proved highly significant as already derived from the analysis with the IFX toolbox (*b* = 0.70, SE = 0.11, *t* = 6.19, *p* < 0.0001). It is possible to report so-called simple effects in multiple regression allowing for interaction effects (see Cohen et al., 2003; Hayes, 2013). In short, simple effects are comparable to main effects in principle, but are conditional on the centering (here: mean-centering) of the remaining predictors in the model (Cohen et al., 2003). Simple effects change significantly as a function of the moderator if there is a significant interaction concerning the predictor of interest. We assessed simple effects for all predictors and found that there was a highly significant simple effect of APOE allele status in rIFG (*t* = −6.26, *p* < 0.001), and—as already pointed out above—a significant simple effect of BrainAGE (*t* = −2.12, *p* = 0.05), but not a significant effect of accuracy. This underlines that the interaction is driven by APOE allele status and is significant only at positive BrainAGE. An overview of simple and interaction effects in our multiple regression model can be found in Supplement 2. Taken together, performance and rIFG activation were positively related at positive BrainAGE scores in e4 carriers and there was no such relationship in non-e4 carriers at any BrainAGE (Figure 3B). With the help of BrainAGE, we were able to explain a greater amount of variance compared to the implementation of CA alone.

For exploratory purposes, we tested the same interaction with an uncorrected threshold (*p* < 0.001). Interestingly, we found a similar interaction in the contralateral homologous area to the above-reported peak in rIFG (lIFG pars orbitalis; MNI x = −28 y = 27 z = −12; *t* = 4.09; *k* = 63; *R*^2^ = 0.59; *p* = 0.01; *R*^2^ change = 0.25, *p* = < 0.001; Cohen's *f*^2^ = 0.61) as well as in left and right middle frontal gyrus (lMFG; MNI x = −34 y = 3 z = 55; *t* = 5.05; *k* = 57; *R*^2^ = 0.72, *p* < 0.001; *R*^2^ change = 0.25, *p* < 0.001; Cohen's *f*^2^ = 0.89; rMFG; MNI x = 38 y = 9 z = 40; *t* = 3.88; *k* = 28; *R*^2^ = 0.59, *p* = 0.01; *R*^2^ change = 0.22, *p* = 0.002; Cohen's *f*^2^ = 0.54). In all three areas, there was a similar activation pattern compared with the peak in rIFG, with APOE ε4 carriers exhibiting increased recruitment with higher BrainAGE together with better performance, with significance regions beginning around the mean BrainAGE of −0.46 years (Figure 3). Hence, the effect is significant predominantly in individuals with positive BrainAGE. Concerning non-ε4 carriers, we identified a negative region of significance in lIFG (Figure 3). Hence, individuals without genetic burden recruited the area less with increasing BrainAGE together with better task performance. The additional areas, although not significant at a corrected threshold when analyzed with SPM, proved to be significant when further tested with the PROCESS macro including CA as a nuisance variable. The three-way interaction of task performance, BrainAGE, and APOE variant remained highly significant in lIFG (*b* = 0.71, SE = 0.19, *t* = 3.78, *p* < 0.0001), lMFG (*b* = 1.53, SE = 0.34, *t* = 4.53, *p* < 0.0001), and rMFG (*b* = 0.87, SE = 0.25, *t* = 3.52, *p* < 0.0001), confirming the incremental benefit of the moderator variables.

The same three-way interaction was tested with CA instead of BrainAGE as a moderator in a previous study of the same sample. At an FWE corrected threshold (*p* < 0.05) as well as at an uncorrected threshold (*p* < 0.001), we did not find a moderating effect of CA and APOE on the relationship between performance and prefrontal activation. A voxel-based morphometry (VBM) analysis of the same sample confirmed that there were no differences in gray matter volume between APOE ε4 and non-ε4 carriers (Scheller et al., 2017), in a way that the identified differential activation is not biased by structural abnormalities.

## Discussion

The aim of the current study was to investigate potential compensatory recruitment in healthy older individuals with a compensation model comprising the components task performance, brain activation and two measures related to pathological burden in aging, BrainAGE, and APOE variant. First, individuals with higher BrainAGE did not require additional neuronal resources to perform a cognitively demanding WM task successfully, hence compensatory brain activation was not detected when BrainAGE alone moderated the relationship between performance and PFC activation. Second, the three-way interaction of performance, namely BrainAGE and APOE variant was examined. Here we found increased activation in bilateral inferior frontal as well as bilateral middle frontal gyrus at higher BrainAGE with better performance, fulfilling one clear-cut criterion of successful compensation (Cabeza and Dennis, 2013). The interaction was driven by ε4 carriers, thus with the combination of a positive BrainAGE and the unfavorable ε4 allele, potential compensatory frontal recruitment in our sample of healthy older adults became apparent. Moreover, the effect remained significant after the variance explained by CA was partialed out.

### Task performance as focal predictor, BrainAGE vs. CA as a moderator

When testing the moderating effects of BrainAGE on the relationship between task performance and prefrontal activation, we did not find significant interaction effects and thus no indication of compensation. Fourteen of thirty-four study participants had an estimated higher BrainAGE compared to their CA. Hence, the majority of our sample exhibited younger-appearing brains, which might be the reason why we did not identify compensatory areas when BrainAGE alone was implemented as a moderator. Younger-appearing brains are associated with higher years of education (Steffener et al., 2016), which is reflected in our highly educated sample drawn from the older population of a university town. The result also conforms to previously obtained findings with CA instead of BrainAGE as a moderator, as we did not find an additional activation in PFC with higher CA in recent work (Scheller et al., 2017). Compensatory patterns might surface in individuals with higher CA/BrainAGE compared to those investigated here, but our sample was presumably too high-functioning due to the above-mentioned selection bias to detect compensatory recruitment with proxies of biological age alone.

### Task performance as focal predictor, BrainAGE vs. CA and APOE variant as moderators

After combining BrainAGE with APOE variant as moderator variables to yield a more detailed characterization of pathological burden, we identified increased prefrontal recruitment compatible with successful neuronal compensation. Specifically, the three-way interaction of task performance, BrainAGE, and APOE variant was significant in rIFG and as revealed by exploratory analyses, also in lIFG and bilateral MFG, with large effect sizes. The increased recruitment of these areas along with better task performance confirms one criterion for successful compensation (Cabeza and Dennis, 2013), such that better performance is associated with higher activation in double-burdened individuals with the ε4 allele and a positive BrainAGE. A second criterion for successful compensation as stated by Cabeza and Dennis, namely the disruption or enhancement of this positive relationship between task performance and brain activation, is not included in the current study. CA did not impact on these interaction effects, as we controlled its influence by implementing CA as a nuisance variable. The results further corroborate previously reported findings on compensatory recruitment in prefrontal cortex. For instance, medial PFC was found to be increasingly activated in ε4 carriers before (Filbey et al., 2010) as well as ventromedial PFC with slightly different fMRI tasks (Wishart et al., 2006). Recent work suggests that the ability to modulate MFG activation (among others) to increasing levels of difficulty in an n-back task is associated with successful cognitive aging (Kennedy et al., 2017).

The negative effect in lIFG observed in non-ε4 carriers might be a sign of processing efficiency, i.e., they perform best when using a concise task-related network without additional areas, as their need for compensation might still be small due to lack of genetic burden (Goh and Park, 2009; Reuter-Lorenz and Park, 2014). Activating additional areas might therefore be a sign dedifferentiation in non-ε4 carriers, which could explain the negative association with task performance (Goh, 2011). Association of lIFG activation with both successful and un-successful compensation depending on genetic burden underlines the importance of distinguishing APOE variants in future studies. The absence of effects compatible with potential compensation in non-ε4 carriers at our chosen statistical threshold does not signify that these individuals do not compensate or are not able to compensate. Due to previous work on a large multicentric sample (Klöppel et al., 2015), we assume that compensation is highly variable across individuals, hence group-level statistics might not be able to grasp such effects. As ε4 carriers and non-ε4 carriers performed equally well in the WM task, we conclude that ε4 needed to recruit additional neuronal resources to reach the same level of accuracy as non-ε4 carriers.

Our compensation-related findings can also be viewed as an elaboration of previous results of the same sample. Prior to the availability of the sample's BrainAGE coefficients, we had already identified bilateral areas on the margin of IFG and insula as compensatory areas in APOE ε4 carriers (Scheller et al., 2017). With the help of BrainAGE, we were able to better stratify these findings by revealing a further association of IFG recruitment with BrainAGE: Not only do APOE ε4 carriers show a need for compensation, but especially do APOE ε4 carriers with older-appearing brains, i.e., individuals with maximal pathological burden. Recent work suggests that APOE ε4 is associated with a different lifespan trajectory regarding the modulation of brain activation under cognitive load (Foster et al., 2017). This corroborates our findings of APOE variant as a strong moderator with high impact on neuronal recruitment.

DLPFC is part of the well-established WM network (Owen et al., 2005; Rottschy et al., 2012) and frontal areas show compensatory activation across several cognitive domains (Cabeza, 2002; Davis et al., 2008; Cabeza and Dennis, 2013), hence we restricted our analysis to frontal cortex. In addition, compensation is assumed to take place in usually task-relevant areas and—when neuronal resources decline—in additional areas often close to the established task network (Reuter-Lorenz and Park, 2014). Thus, we can assume that increased activation in inferior as well as middle parts could have buffered beginning deficits in DLPFC.

Why we observe a significant interaction of task performance, BrainAGE, and APOE and not of task performance, CA, and APOE cannot be determined unambiguously, as our sample size was not sufficient for more complex statistical models, i.e., a four-way interaction of task performance, CA, BrainAGE, and APOE variant. A larger sample or a replication sample would be desirable to strengthen our findings. Still, taking brain structure into account when approximating pathological burden helped to obtain a more fine-grained picture of compensatory recruitment. Consequently, we would argue that our ε4 carriers showed compensation in PFC, but that potential compensatory recruitment could only be revealed when taking into account a combination of risk factors, i.e., the most accurate approximation of pathological burden available. Moreover, our cross-sectional design only captures inter-individual variability. To follow individuals' compensation trajectories, i.e., the initialization and further development of compensatory recruitment, longitudinal investigations are needed (Nyberg et al., 2010; Gregory et al., 2017). Finally, to strengthen the reported findings, future work will need to prove that also a second criterion of successful compensation (Cabeza and Dennis, 2013) is fulfilled, namely the disruption or enhancement of the identified positive relationship between task performance and brain activation by e.g., NIBS procedures.

## Conclusion

BrainAGE together with APOE variant has proved a helpful proxy of pathological burden to be implemented in models of neuronal compensation. The suggested combination of structural and functional imaging as well as genetic data translating theoretical frameworks to statistical models of compensation should be transferred to other cognitive domains as well as further samples of healthy older individuals and patients with beginning neurodegenerative disease. As proposed in a review of studies on compensation (Scheller et al., 2014), previous results could be revisited with the same model of compensation as suggested here. Structural imaging and herewith the opportunity to compute BrainAGE is easily available in functional imaging studies and thus key compensatory regions of specific cognitive functions could be identified, further characterized and potentially amplified by non-invasive brain stimulation combined with cognitive training programs.

## Ethics statement

This study was carried out in accordance with the recommendations of the ethics committee of the Albert Ludwigs University Freiburg with written informed consent from all subjects. All subjects gave written informed consent in accordance with the Declaration of Helsinki. The protocol was approved by the ethics committee of the Albert Ludwigs University Freiburg.

## Author contributions

ES, JP, LS, CK, and SK: designed the study; JP, JL, and LS: acquired data; ES, CK, and CG: analyzed data; ES, JW, and SK: interpreted data for the study; JW and ES: created figures; ES and SK: drafted the manuscript and all authors revised it critically for important intellectual content. All authors gave their final approval of the version to be published and agree to be accountable for all aspects of the work.

### Conflict of interest statement

The authors declare that the research was conducted in the absence of any commercial or financial relationships that could be construed as a potential conflict of interest.
