# Supplementary material for: Brain Aging and APOE ε4 Interact to Reveal Potential Neuronal Compensation in Healthy Older Adults
Source: Front Aging Neurosci. 2018 Mar 20;10:74. doi: 10.3389/fnagi.2018.00074 (PMC5869204; doi:10.3389/fnagi.2018.00074)
Supplement: Supplementary file 1 [file Table1.DOCX]

Supplement 1

Classification of all 34 participants according to their APOE ε4 allele status as well as their brainAGE index.

| participant number | APOE ε4 (ε4 / non- ε4) | brainAGE index |
| --- | --- | --- |
| 1 | non- ε4 | -5,75 |
| 2 | non- ε4 | 1,33 |
| 3 | non- ε4 | 5,49 |
| 4 | non- ε4 | 2,67 |
| 5 | non- ε4 | -3,02 |
| 6 | non- ε4 | -3,65 |
| 7 | non- ε4 | 3,44 |
| 8 | non- ε4 | -1,94 |
| 9 | non- ε4 | 3,77 |
| 10 | non- ε4 | -1,43 |
| 11 | non- ε4 | -8,29 |
| 12 | non- ε4 | -3,65 |
| 13 | non- ε4 | -7,15 |
| 14 | non- ε4 | ,09 |
| 15 | non- ε4 | -,28 |
| 16 | non- ε4 | 1,72 |
| 17 | non- ε4 | 1,68 |
| 18 | non- ε4 | -2,70 |
| 19 | non- ε4 | -2,50 |
| 20 | non- ε4 | 1,02 |
| 21 | non- ε4 | -2,53 |
| 22 | ε4 | 6,84 |
| 23 | ε4 | 3,95 |
| 24 | ε4 | 10,00 |
| 25 | ε4 | -4,57 |
| 26 | ε4 | -,85 |
| 27 | ε4 | 1,78 |
| 28 | ε4 | -,61 |
| 29 | ε4 | -4,65 |
| 30 | ε4 | -1,42 |
| 31 | ε4 | -1,93 |
| 32 | ε4 | -4,70 |
| 33 | ε4 | -3,52 |
| 34 | ε4 | 5,63 |
