# Supplementary material for: Brain Aging and APOE ε4 Interact to Reveal Potential Neuronal Compensation in Healthy Older Adults
Source: Front Aging Neurosci. 2018 Mar 20;10:74. doi: 10.3389/fnagi.2018.00074 (PMC5869204; doi:10.3389/fnagi.2018.00074)

Supplement 2:

Overview of regression coefficients in right inferior frontal gyrus. First, a model summary with the respective regression coefficient R^2^ (R-sq) is listed. Then, all regression coefficients including simple and interaction effects are depicted. Unstandardized regression coefficients (coeff), their standard error (se) as well as corresponding t- and p- values and lower and upper bounds of the confidence intervals (LLCI and ULCI) are reported. The nomenclature of the reported interactions can be found below the model description. Finally, the R^2^ increase due to the inclusion of the three-way-interaction is reported.

Note: BA_GMWM = BrainAGE; acc2b = accuracy; APOE_01 = APOE allele status; educ = years of education; Age = chronological age


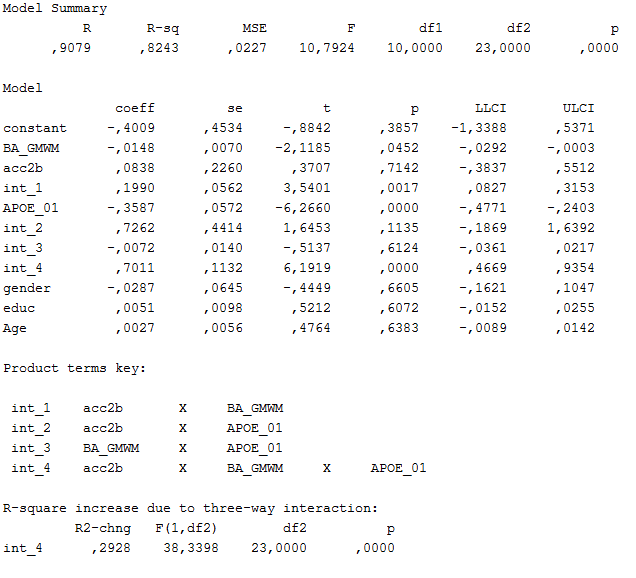

Supplement: Supplementary file 2 [file Table2.DOCX]
